# Using GeneReg to construct time delay gene regulatory networks

**DOI:** 10.1186/1756-0500-3-142

**Published:** 2010-05-25

**Authors:** Tao Huang, Lei Liu, Ziliang Qian, Kang Tu, Yixue Li, Lu Xie

**Affiliations:** 1 School of Life Sciences, Fudan University, Shanghai 200433, PR China; 2 Key Laboratory of Systems Biology, Shanghai Institutes for Biological Sciences, Chinese Academy of Sciences, Shanghai 200031, PR China; 3 Shanghai Center for Bioinformation Technology, Shanghai 200235, PR China; 4 National Heart, Lung, and Blood Institute (NHLBI), NIH, MD 20892, USA; 5 Innovation Center China, AstraZeneca, Shanghai 201203, PR China

## Abstract

**Background:**

Understanding gene expression and regulation is essential for understanding biological mechanisms. Because gene expression profiling has been widely used in basic biological research, especially in transcription regulation studies, we have developed GeneReg, an easy-to-use R package, to construct gene regulatory networks from time course gene expression profiling data; More importantly, this package can provide information about time delays between expression change in a regulator and that of its target genes.

**Findings:**

The R package GeneReg is based on time delay linear regression, which can generate a model of the expression levels of regulators at a given time point against the expression levels of their target genes at a later time point. There are two parameters in the model, time delay and regulation coefficient. Time delay is the time lag during which expression change of the regulator is transmitted to change in target gene expression. Regulation coefficient expresses the regulation effect: a positive regulation coefficient indicates activation and negative indicates repression. GeneReg was implemented on a real Saccharomyces cerevisiae cell cycle dataset; more than thirty percent of the modeled regulations, based entirely on gene expression files, were found to be consistent with previous discoveries from known databases.

**Conclusions:**

GeneReg is an easy-to-use, simple, fast R package for gene regulatory network construction from short time course gene expression data. It may be applied to study time-related biological processes such as cell cycle, cell differentiation, or causal inference.

## Background

With the rapid development of microarray technology, more and more short time course gene expression data have been generated; with such abundant high-throughput screening data available, researchers have tried to infer, or reverse-engineer, gene networks. In general, the existing models for network inference can be grouped into three categories: logical models, continuous models and single-molecule level models [1]. Logical models such as Boolean networks and Petri nets could represent the network structure but are unable to describe dynamic processes. While single-molecule level models such as stochastic simulation algorithm could provide high resolution modeling and analysis, but only on limited molecules with well-known reactions among them. Single-molecule level models are not suitable for large scale regulatory network reconstruction. There were several widely-used general algorithms for network inference, such as information-theoretic approaches, Bayesian-based models, and ordinary differential equations [2]. Many of them belong to the continuous models. There may be other models which could integrate prior knowledge to improve the performance, but we only considered the *ab initio *network inference approaches here as prior knowledge is able to be integrated into most *de novo *network reconstruction methods easily.

The most well known software of information-theoretic approaches for gene network inference is ARACNE [3,4]. The information-theoretic approach was first proposed by Butte and Kohane [5], with their relevance network algorithm. Information-theoretic approaches use mutual information to compare expression profiles from a set of microarrays. The definition of mutual information requires each experiment to be statistically independent from the others. Thus, information-theoretic approaches can deal with steady-state gene expression data or with time-series data given that the sampling interval is long enough to assume that each point is independent of the previous points. This assumption, however, does not hold for most biological time series datasets, because the interval between measured time points is usually short. In many cases, biologists actually want to see the connections between events happening at an earlier time point and those at a later one, rather than looking at isolated time points.

Banjo is a representative gene network inference software based on Bayesian network formalism [6]. Because Banjo implements both Bayesian and dynamic Bayesian networks, it can infer gene networks from steady-state gene expression data or from time-series gene expression data. In Banjo, heuristic approaches are used to search the network space to find the network graph G, which requires large datasets in which the number of genes is much smaller than the number of experiments. In most gene expression datasets, however, the number of genes is much larger than the number of experiments.

Ordinary differential equations (ODEs) based reverse-engineering algorithms relate changes in gene transcript concentration to each other and to an external perturbation. To reverse-engineer a network using ODEs requires selection of an ODE function and estimation of unknown parameters from gene expression data using some optimization technique. ODE-based approaches yield directed graphs and can be applied to both steady-state and time-series expression profiles, but they are often very complex and slow, and do not provide insight into the biological meanings of each parameter.

To address the limitations of existing approaches for gene regulatory network construction from short time course gene expression data, we have developed an easy-to-use, simple, and fast R package: GeneReg. GeneReg is based on a time delay linear regression model, which is similar to Kim's ordinary differential equation[7]. The function used in this model, however, is a linear function with two parameters, time delay and the regulation coefficient. Time delay is the time required to transmit change in regulator gene expression to change in target gene expression. The regulation coefficient represents the regulation effect: a positive coefficient indicates activation and negative indicates repression.

The most important improvement of our model was the time delay of each regulator can be exactly calculated and different regulators could have different time delays. Time delay is an important concept in biological regulatory mechanisms, especially for transcription factors. As we known, transcription factor could only regulate its target genes in protein form but in microarrays studies, the measured abundance of transcription factor is its mRNA expression level. The mRNA of transcription factor must be translated into protein and then the proteins of transcription factor regulate the expression of downstream genes. There is a time delay from the mRNA of transcription factor being generated to the actual regulation of transcription factor. What's more, time delay is an almost unmeasureable variable by traditional experiments as there were too many unclear processes during translation and the factors which could affect the process were unpredictable. The time delay calculated in our model provided a higher level estimation of this important but unmeasureable biological variable.

The biological meanings of the two parameters in our model are both clear and important for understanding gene regulatory mechanisms. As the linear model is simple and the forward selection optimization of parameters is easy to compute, GeneReg is much faster than similar software and could be used in deciphering genome-wide gene regulatory networks. Our models do not require prior knowledge about regulatory mechanisms, although prior knowledge could be integrated, for example if certain regulators were already known to regulate the target gene, they could be added into the model first. The model with time delay and regulation coefficient can be used to obtain qualitative insights about regulatory networks and discovery novel regulations. This linear model assumption may ignore parts of the nonlinear regulations, but it allows a high level of abstraction and efficient inference of network structure and regulation functions. When higher resolution to detailed regulatory relationship is desired, the linear model can be replaced with more complex nonlinear models, such as mass action models or Hill models[8].

GeneReg was implemented on a real Saccharomyces cerevisiae cell cycle dataset. The results were found to reflect known dynamic expression profiles, with 32.45% of the regulations modeled in wild type cells and 32.61% of regulations in cyclin mutant cells consistent with what can be found in the YEASTRACT and/or STRING databases. These are fairly good results, considering that our large scale gene network construction was only based on a single small time series gene expression dataset [9].

## Results and Discussion

### Time delay linear regression model

The model is based on a linear regression of the expression levels of regulators at time *t *- Δ*t *against the expression level of their target genes at time *t*. Δ*t *is the time delay between expression of transcription factors and expression of downstream genes, and can differ from gene to gene.

Suppose we have a set of time course data covering time points *T*_1_, *T*_2_,..., *T*_*k*_, and target gene *g *is regulated by n regulators *tf*_1_, *tf*_2_,..., *tf*_*n *_in a linear manner. These relationships can be formulized as below:(1)

where  (*g*) is the relative expression level of target gene *g *at time point *t*_*g *_to the baseline,  is the relative expression level of *tf*_*i *_at time point *t*_*g *_- Δ*t*_*i*_, Δ*t*_*i*_, is the time delay of *tf*_*i*_'s regulation to gene g, and *a*_*i *_is the regression coefficient of *tf*_*i*_.

## Computational algorithm

Our method aims to select a set of possible regulators with certain time delays to estimate the dynamic expression pattern of a target gene. The method uses an AIC (Akaike information criterion) [10] model selection criteria with forward selection to iteratively add possible regulators from the candidate pool. AIC, which describes the tradeoff between model complexity and the estimated residual variance, is defined as(2)

where *k*_*p *_is the number of parameters in the statistical model, and *L *is the maximized value of the likelihood function for the estimated model. AIC increases as the number of parameters increase and decreases as the residual variance decreases. The smaller AIC indicates better model.

In practical computation, it is time-consuming to compute the AIC statistics for all possible regression models. Forward selection method [11] was used to avoid the complexity of exhaustive search. As one of the greedy optimization method, in forward selection, a variable once included can never be removed [11]. The model optimized by forward selection method may be not the best, but it is an acceptable compromise with fast speed and good performance. What's more, in specific condition, the regulators of one target gene couldn't be as much as the database suggested, or as complex as the mixture of all known mechanism and identifying the key regulators first will at least guarantee that the major regulations wouldn't be missed.

The model's explanatory ability is evaluated by adjusted R^2 ^[12]. Adjusted R^2 ^is an improved R^2 ^that adjusts for the number of explanatory terms in a model. The adjusted R^2 ^is defined as(3)

where *n*_*r *_is the number of regulators in the linear model, and *k*_*s *_is sample size

The computational procedure of our method can be summarized as following:

1) Sorting the regulators based on their relevance with target gene

The goal of this procedure was to filter the irrelevant regulators first and sort the regulators according to their importance to the regulation. To each regulator, all possible time delays were traversed and the adjusted R^2 ^of best single regulator regression model with smallest AIC was calculated. Regulators didn't meet a pre-specified adjusted R^2 ^cutoff were considered as irrelevant with the target gene and were filtered. The left *M *regulators were sorted according to their smallest AICs.

After the pre-evaluation procedure, a regulator set *S *is provided:(4)

The index reflects the evaluations for regulator. For example, If a < b, *tf*_*a *_has smaller AIC than *tf*_*b *_and *tf*_*a *_is considered to be better than *tf*_*b*_.

2) Regulation model optimization with forward selection of regulators and time delays

**Figure 1:**
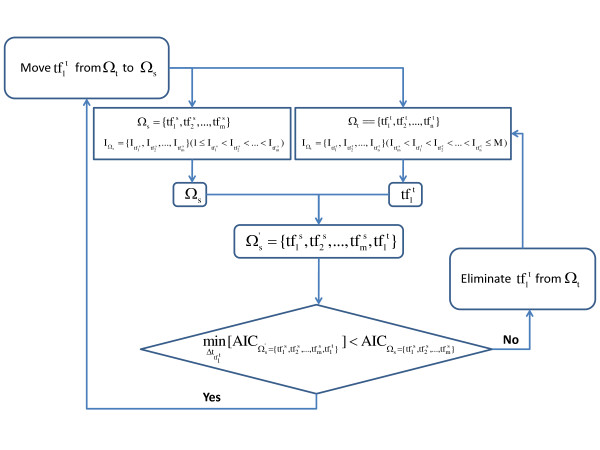
**The forward selection of regulators in time delay linear regression model**. The selected regulator set with *m *regulators is defined as  the *S *indexes of regulators in Ω_s _are  The to-be-selected regulator set with *n *regulators is defined as  and the *S *indexes of regulators in Ω_*t *_are  In the forward selection procedure, each regulator in Ω_*t *_will be successively tested whether it can be added into Ω_*s*_. During each test process, the time delays of Ω_*s *_are held the same. All possible time delays of the regulator in Ω_*t *_with smallest *S *index that is  are traversed, and the time delay of  which achieves the smallest AIC of  is considered as the optimal time delay of . This smallest AIC of  is then compared with the AIC of original . If  wil be moved from Ω_*t *_to Ω_*s*_. Otherwise,  will be eliminated from Ω_*t*_. In next round, the new regulator in Ω_*t *_with smallest *S *index will be tested until Ω_*t *_is empty.

Step one can only provide a list of relevant regulators by sorting them according to their importance to the regulation, but it is still unknown which fore regulators in the list should be selected to establish the time delay regression model. The best fore regulators are selected by testing all possible top regulator sets, and choosing the regulator set that can achieve the smallest AIC with forward selection. The forward selection procedure was illustrated in Figure 1.

The possible regulator subset *s*_*i *_can be expressed using the following equation:(5)

The initial regulator subset is .

The selected regulator set with *m *regulators is defined as , the *S *indexes of regulators in Ω_*s *_are . The to-be-selected regulator set with *n *regulators is defined as  and the *S *indexes of regulators in Ω_*t *_are .

In the forward selection procedure [13], each regulator in Ω_*t *_will be successively tested whether it can be added into Ω_*s*_. During each test process, the time delays of Ω_*s *_are held the same. All possible time delays of the regulator in Ω_*t *_with smallest *S *index that is  are traversed, and the time delay of  which achieves the smallest AIC of  is considered as the optimal time delay of . This smallest AIC of  is then compared with the AIC of original . If  will be moved from Ω_*t *_to Ω_*s*_. Otherwise,  will be eliminated from Ω_*t*_. In next round, the new regulator in Ω_*t *_with smallest *S *index will be tested until Ω_*t *_is empty.

After all *M *- 1 regulators have been tested to add into the initial regulator subset  the optimized time delay regression model was established which not only have the optimal regulators but also their corresponding optimal time delays. At the end of the whole process, the adjusted R^2 ^of this final optimized model was calculated. If the adjusted R^2 ^meets pre-specified criteria, it suggests the optimized time delay model could explain this target gene's expression pattern and is applicable. Otherwise, this target gene's expression pattern can't be explained by our time delay linear regression model and maybe other more complex nonlinear models are needed.

## Implementation

### Yeast cell cycle dataset

To evaluate our approach on biological time course gene expression data, we applied the method to Saccharomyces cerevisiae cell cycle data publicly available at GEO http://www.ncbi.nlm.nih.gov/geo with accession number GSE8799. The dataset includes the gene expression profiles of wild type cells and cyclin mutant cells at 15 time points during two cell cycles and each genotype has two replicates with different 15 time points on life line. In our study, we merged the two replicates of each genotype based on their life lines and then there were 30 time points for wild type cells and cyclin mutant cells. 1271 periodic genes which were defined in Orlando's work[14] and considered as cell cycle related genes formed the list of target genes and their regulations were analyzed. A candidate pool of potential regulators which included 35 transcription factors was constructed by intersecting the 1271 periodic genes with all transcription factors in the YEASTRACT database [15,16]http://www.yeastract.com/.

### Time delay linear model

First, the data were transformed to a log_2 _ratio scale. The expression level of the first time point was taken as baseline. The gene expression level at each time point subtracted the baseline to get the relative expression level. Then B spline interpolation [17] was applied to expand the original 30 time points to 100 time points.

Time delay linear models then were constructed based on the interpolated expression data and candidate pool of regulators, with the adjusted R^2 ^cutoff for single regulator regression and multiple regulator regression set at 0.8 and 0.9. Additional files 1 and 2 give the time delay models of wild type cells and cyclin mutant cells, respectively.

**Figure 2:**
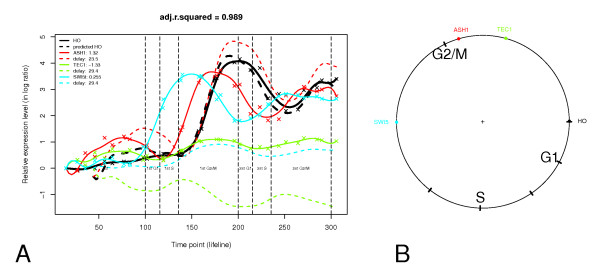
**The time delay linear regression model of the *HO *gene in wild type cells**. (A) The expression pattern of *HO *and its regulators *ASH1*, *TEC1*, and *SWI5*. The solid lines represent interpolated values based on experimentally observed values (X symbol), and the black dotted line represents the values of the target gene fitted by the model. The colored dotted lines represent the regulatory contribution of each regulator; the black dotted line is the sum of these. The time delays of *ASH1*, *TEC1*, and *SWI5 *are 23.5, 29.4, and 29.4 minutes, respectively. Before 95.6 min, the cell cycle phase is recovering from synchrony; from 95.6 to 107.5 min, the phase is 1^st ^cycle G1; from 107.5 to 122.9 min, the phase is 1st cycle S; from 122.9 to 172.5 min, the phase is 1st cycle G2/M; from 172.5 to 184.4 min, the phase is 2nd cycle G1; from 184.4 to 199.8 min, the phase is 2nd cycle S; from 199.8 to 249.3 min, the phase is 2nd cycle G2/M. (B) Cell cycle diagram of corresponding model. The target gene *HO *and its regulators are arranged on the basis of the time of peak transcript levels.

As an example, Figure 2 shows the time delay linear regression model of the *HO *gene in wild type cells. *HO *encodes an endonuclease responsible for initiating mating-type switching, a process in which MATa cells change to MATalpha cells or vice versa. This process is controlled by Swi4p-Swi6p, Swi5p, and Ash1p according to the Saccharomyces Genome Database (SGD) [18]. In our model, we found *ASH1*, *TEC1*, and *SWI5 *to be the mostly likely regulators of *HO*.

Finally, the whole regulatory network was plotted based on the series of time delay linear models. Additional files 3 and 4 give the time delay network of wild type cells and cyclin mutant cells, respectively.

### Comparison with YEASTRACT and STRING

To evaluate the performance of the gene regulatory networks constructed based on time delay linear models, we generated a reference network from the YEASTRACT [15,16] and STRING [19] databases. YEASTRACT is a curated database of regulatory associations between transcription factors and target genes in Saccharomyces cerevisiae, based on the literature. STRING is a database that quantitatively integrates direct (physical) and indirect (functional) associations from different sources. We found that 32.45% of our modeled regulations in wild type cells and 32.61% of regulations in cyclin mutant cells were consistent with YEASTRACT or STRING, a fair result for large-scale gene network construction based on a single small gene expression dataset [9].

The above prediction accuracies were calculated based on the data presented in additional files 5 and 6. The last two columns of each file compare the modeled regulations in wild type (Additional file 5) and cyclin mutant (Additional file 6) cells against data found in YEASTRACT or STRING. In these columns, 1 indicates consistency with the database, 0 indicates inconsistency when the regulator and target gene are included in the database, and NA indicates the database does not include the regulator or target gene. Regulations consistent with either the YEASTRACT database or STRING database (1 in either column) were considered as true, while regulations consistent or inconsistent with either the YEASTRACT database or STRING database (0 or 1 in either column) were considered as total regulations. (Regulations with NA in both the YEASTRACT database and STRING database were excluded from accuracy calculation.) Prediction accuracy was defined as the number of true regulations divided by the number of total regulations.

**Figure 3:**
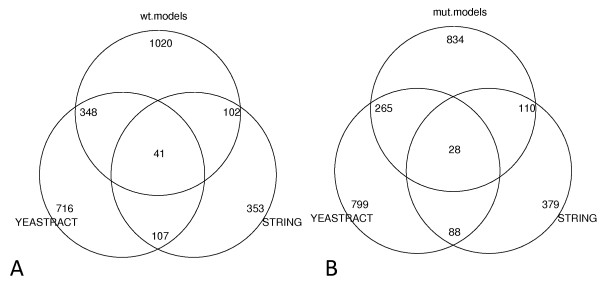
**Overlap of predicted ORF regulations with YEASTRACT and STRING databases**. (A) The overlap of predicted ORF regulations in wild type cells with YEASTRACT and STRING. (B) The overlap of predicted ORF regulations in cyclin mutant cells with YEASTRACT and STRING.

The above comparison was on the probe level. Figure 3 shows the overlap of total predicted regulations with YEASTRACT and STRING-documented regulations at the open reading frame (ORF) level in both wild type cells and cyclin mutant cells.

The activity of transcription factors is an important factor for gene regulation. As many transcription factors are post-translationally controlled, their activity cannot always be observed directly by measuring changes in their mRNA expression level. Additionally, certain condition-specific regulators vary with different perturbations; for example, many regulatory relationships differ between wild type and cyclin mutant cell networks. All of these issues affect the overlap of our network with that documented in the known databases. In general, there is no final or best network, only a group of possible networks that are nearly equally useful.

### Comparison with regulatory networks without time delay

To better understand the impact of time delay in network construction, we build the networks without time delay in wild type cells and cyclin mutant cells by setting the parameter of our program, max time delay as 0. When time delay was not considered, there were only 3050 and 2489 predicted regulations in wild type cells and cyclin mutant cells under the same criteria; 32.40% of predicted regulations in wild type cells and 31.94% of predicted regulations in cyclin mutant cells were consistent with YEASTRACT or STRING. As shown in additional files 5 and 6, if time delay is considered, the number of predicted regulations will increase 54.65% and 39.25%, to 4717 and 3466 in wild type cells and cyclin mutant cells, respectively. And the percentages of known regulations in the time delay considered networks in wild type cells and cyclin mutant cells were 32.45% and 32.61%, slightly higher than the networks without time delay. Our results suggest that considering time delay in network construction will increase the number of predicted regulations, but not increase the false positive rate.

### Time delay network of eight well known transcription factors

**Figure 4:**
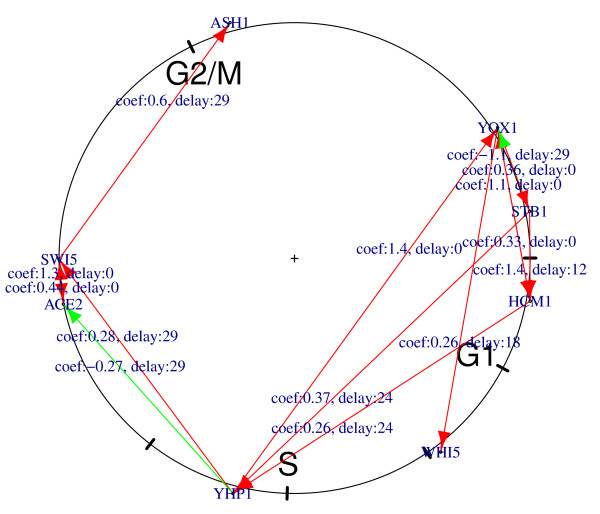
**Time delay network of eight well known transcription factors for wild type cells**. Eight well known transcription factors (YOX1, STB1, HCM1, WHI5, YHP1, ACE2, SWI5, and ASH1) were specifically investigated based on the time delay network of wild type cells. The genes are arranged based on time of peak transcript levels. A red arrow indicates positive regulation; green indicates negative regulation.

Eight well known transcription factors (YOX1, STB1, HCM1, WHI5, YHP1, ACE2, SWI5, and ASH1) studied in Orlando's work[14] were specifically investigated based on the time delay network of wild type cells. Figure 4shows the time delay network of these eight transcription factors in wild type cells. Table 1 gives the detailed parameters calculated in the time delay models. The predicted network consists of 14 regulations, eight of which find support in YEASTRACT, STRING or David Orlando's study [14]. The other six regulations might be minor effects resulting from the major regulator's co-factors.

**Table 1:** The time delay models of eight well known transcription factors for wild type cells

Regulator	Target	Coef	Delay	Adj.R. Squared	YEASTRACT	STRING	**Orlando et al. **[14]
							
SWI5_YDR146C_1770349_at	ASH1_YKL185W_1772030_at	0.594772	29.40695	0.998474	SWI5->YKL185W	YDR146C <->YKL185W	SWI5->ASH1
YHP1_YDR451C_1778368_at	SWI5_YDR146C_1770349_at	0.27633	29.40695	0.998062	NA	NA	NA
ACE2_YLR131C_1771312_at	SWI5_YDR146C_1770349_at	1.322992	0	0.998062	NA	YLR131C <->YDR146C	NA
SWI5_YDR146C_1770349_at	ACE2_YLR131C_1771312_at	0.440266	0	0.996918	NA	YDR146C <->YLR131C	NA
YHP1_YDR451C_1778368_at	ACE2_YLR131C_1771312_at	-0.27086	29.40695	0.996918	NA	NA	NA
HCM1_YCR065W_1772793_at	YHP1_YDR451C_1778368_at	0.264364	23.52556	0.997296	NA	YCR065W <->YDR451C	NA
STB1_YNL309W_1771976_at	YHP1_YDR451C_1778368_at	0.37007	23.52556	0.997296	NA	NA	NA
HCM1_YCR065W_1772793_at	YOX1_YML027W_1775720_at	1.119924	0	0.998627	NA	YCR065W <->YML027W	NA
STB1_YNL309W_1771976_at	YOX1_YML027W_1775720_at	-1.06256	29.40695	0.998627	NA	NA	NA
YHP1_YDR451C_1778368_at	YOX1_YML027W_1775720_at	1.436395	0	0.998627	NA	YDR451C <->YML027W	NA
STB1_YNL309W_1771976_at	HCM1_YCR065W_1772793_at	1.373068	11.76278	0.997623	NA	NA	STB1->HCM1
YOX1_YML027W_1775720_at	HCM1_YCR065W_1772793_at	0.329105	0	0.997623	NA	YML027W <->YCR065W	NA
YOX1_YML027W_1775720_at	STB1_YNL309W_1771976_at	0.364278	0	0.985918	NA	NA	NA
YOX1_YML027W_1775720_at	WHI5_YOR083W_1772753_at	0.261895	17.64417	0.995457	NA	NA	NA

## Conclusions

As expression profiling technology has grown in popularity, much effort has been devoted to building gene regulation networks based on the wealth of profiling data generated. In this contribution, a new method is proposed to not only construct dynamic gene regulatory networks, but also to calculate the time delays between regulators and downstream genes. Time delay between transcription factor activation/repression and that of its target genes has long been suspected. Our tool allows a visualization of exact time delays, calculated from real in vivo data. Our approach can be applied to investigate important time-related biological processes, such as the cell cycle, cell differentiation and development. Similarly such a method may be important for researchers studying the mechanisms of specific transcription factors, their pathways, and possible interventions for associated diseases.

## Methods

### How to run GeneReg on the example dataset

Time delay regression models can be easily constructed using the R package GeneReg, which is freely available from CRAN http://cran.r-project.org/web/packages/GeneReg/index.html. Additional file 7 contains GeneReg version 1.1.1. Additional file 8 is the R code for the above analysis. The processed example data mentioned above is contained within the R package. We detail the usage of GeneReg on the time course gene expression profiles of wild type cells in the steps below. The analysis of cyclin mutant cells was similar.

(1) B spline interpolation [17] was applied to estimate the expression of 100 time points according to the original data of 30 time points.

data(wt.expr.data)

wt.bspline.data < - ts.bspline(wt.expr.data, ts.point= as.numeric(colnames(wt.expr.data)), data.predict = 100)

(2) A series of time delay linear models were constructed based on the interpolated expression data. Detailed explanation of each parameter in the following code can be found in the help document of the GeneReg package at the above website.

data(tf.list)

wt.models < -timedelay.lm.batch(bspline.data = wt.bspline.data, expr.data = wt.expr.data, regulator.list = tf.list, target.list = rownames(wt.bspline.data), single.adj.r.squared = 0.8, multiple.adj.r.squared = 0.9, maxdelay = ncol(wt.bspline.data)*0.1, min.coef = 0.25, max.coef = 4, output = T, topdf = T, xlab = 'Time point (lifeline)', ylab = 'Relative expression level (in log ratio)')

(3) The whole network was plotted based on the series of time delay linear models constructed in step (2).

plot.GeneReg(wt.models,vertex.size = 2,layout = layout.fruchterman.reingold)

## Availability and requirements

• **Project name: **GeneReg

• **Project home page: **http://cran.r-project.org/web/packages/GeneReg/index.html

• **Operating systems: **Platform independent

• **Programming language: **R

• **Other requirements: **GeneReg depends on two other R packages: splines and igraph

• **License: **LGPL

• **Any restriction to use by non-academics: **none

## Abbreviations

(ODE): Ordinary differential equation; (AIC): Akaike Information Criterion; (ORF): open reading frame.

## Competing interests

The authors declare that they have no competing interests.

## Authors' contributions

TH, KT and ZQ carried out the study. TH and LL wrote the manuscript. LX and YL supervised the project. All authors read and approved the final manuscript.

## Supplementary Material

Additional file 1 **Time delay models in wild type cells**. Each file is a time delay model of one target gene. Click here for file

Additional file 2 **Time delay models in cyclin mutant cells**. Each file is a time delay model of one target gene. Click here for file

Additional file 3 **Time delay network of wild type cells**. The time delay network of wild type cells includes 4717 predicted regulations. Click here for file

Additional file 4 **Time delay network of cyclin mutant cells**. The time delay network of cyclin mutant cells includes 3466 predicted regulations. Click here for file

Additional file 5 **Collection of time delay models in wild type cells**. Each row represents one predicted regulation. The seven columns show regulator, target gene, regression coefficient, time delay, adjusted R^2^, consistency with the YEASTRACT database, and consistency with the STRING database. In these last two columns, 1 indicates consistency with the database, 0 indicates inconsistency when the regulator and target gene are included in the database, and NA indicates the database does not include the regulator or target gene. Regulations consistent with either the YEASTRACT database or STRING database (1 in either column) were considered as true, while regulations consistent or inconsistent with either the YEASTRACT database or STRING database (0 or 1 in either column) were considered as total regulations. Regulations with NA in both the YEASTRACT database and STRING database were excluded from accuracy calculation. Click here for file

Additional file 6 **Collection of time delay models in cyclin mutant cells**. Each row represents one predicted regulation. The seven columns show regulator, target gene, regression coefficient, time delay, adjusted R^2^, consistency with the YEASTRACT database, and consistency with the STRING database. In these last two columns, 1 indicates consistency with the database, 0 indicates inconsistency when the regulator and target gene are included in the database, and NA indicates the database does not include the regulator or target gene. Regulations consistent with either the YEASTRACT database or STRING database (1 in either column) were considered as true, while regulations consistent or inconsistent with either the YEASTRACT database or STRING database (0 or 1 in either column) were considered as total regulations. Regulations with NA in both the YEASTRACT database and STRING database were excluded from accuracy calculation. Click here for file

Additional file 7 **Zip file of GeneReg version 1.1.1**. Processed example data are contained within the package. Click here for file

Additional file 8 **R code for analysis**. R code for analysis of wild type cells and cyclin mutant cells. Click here for file
