# Supplementary material for: Using GeneReg to construct time delay gene regulatory networks
Source: BMC Res Notes. 2010 May 25;3:142. doi: 10.1186/1756-0500-3-142 (PMC2892504; doi:10.1186/1756-0500-3-142)
Supplement: Additional file 7 — Zip file of GeneReg version 1.1.1. Processed example data are contained within the package. [file 1756-0500-3-142-S7.ZIP › GeneReg/html/00Index.html]

R: Construct time delay gene regulatory network

# Construct time delay gene regulatory network

---

## Documentation for package ‘GeneReg’ version 1.1.1

## Help Pages

|  |  |
| --- | --- |
| GeneReg | Construct time delay gene regulatory network |
| mut.expr.data | Time course cell cycle dataset of cyclin-mutant cells |
| plot | Plot gene regulatory network |
| plot.GeneReg | Plot gene regulatory network |
| tf.list | The candidate pool of potential regulators |
| timedelay.lm | time delay linear model |
| timedelay.lm.batch | time delay linear model batch |
| timedelay.univariate.lm | Internal function for time delay linear model |
| ts.bspline | B spline interpolation |
| wt.expr.data | Time course cell cycle dataset of wild-type cells |
