# Supplementary material for: Using GeneReg to construct time delay gene regulatory networks
Source: BMC Res Notes. 2010 May 25;3:142. doi: 10.1186/1756-0500-3-142 (PMC2892504; doi:10.1186/1756-0500-3-142)

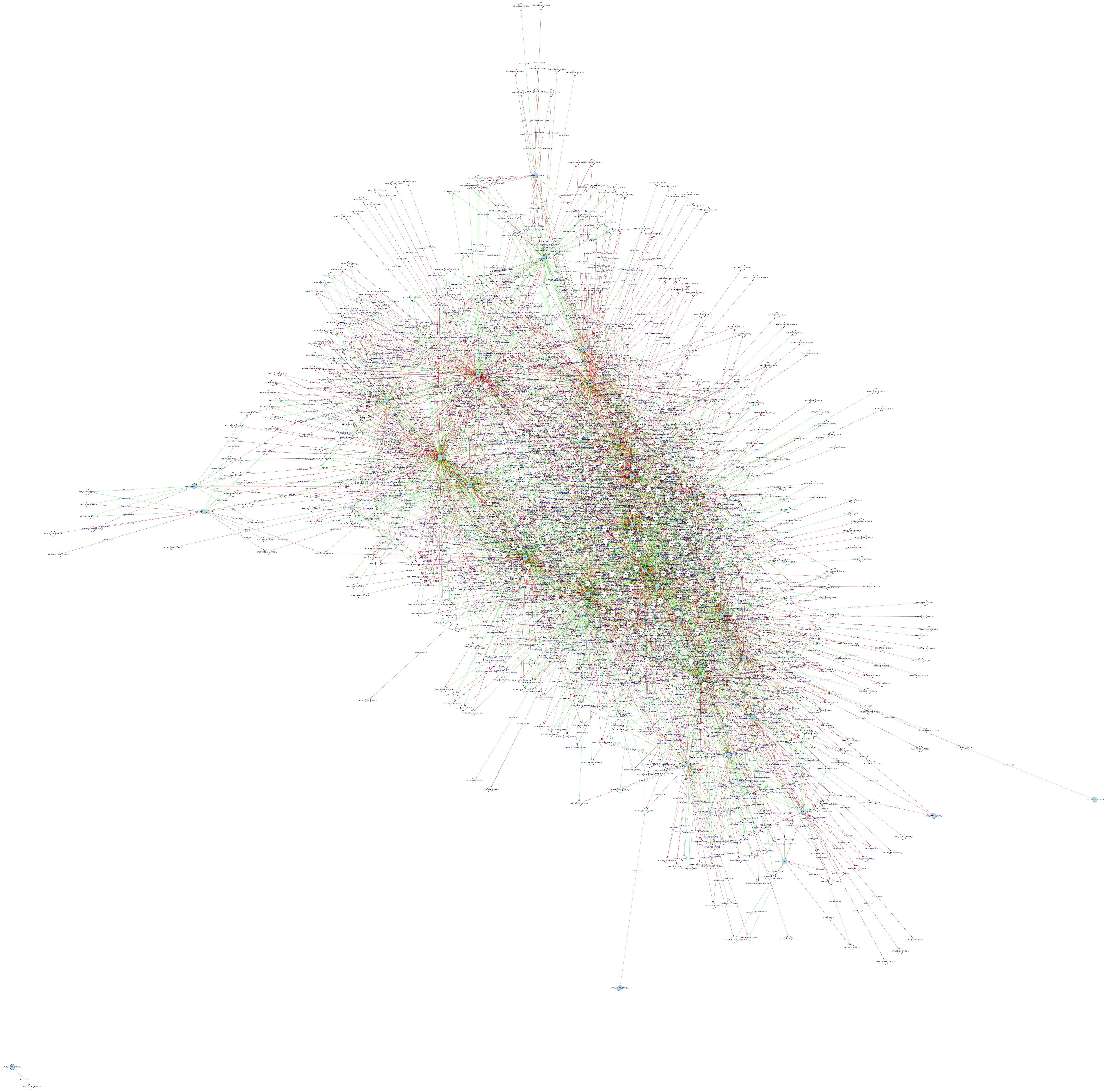

Supplement: Additional file 4 — Time delay network of cyclin mutant cells. The time delay network of cyclin mutant cells includes 3466 predicted regulations. [file 1756-0500-3-142-S4.PDF]
